# One-year outcomes of a bilateral randomised prospective clinical trial comparing PRK with mitomycin C and LASIK

**DOI:** 10.1136/bjo.2008.152579

**Published:** 2009-11-17

**Authors:** A D Wallau, M Campos

**Affiliations:** Vision Institute, Federal University of São Paulo Department of Ophthalmology, São Paulo, Brazil

## Abstract

**Aim:**

To compare 1-year follow-up results of photorefractive keratectomy (PRK) with mitomycin C (MMC) and laser in situ keratomileusis (LASIK) for custom correction of myopia.

**Methods:**

Eighty-eight eyes of 44 patients with moderate myopia were randomised to PRK with 0.002% MMC for 1 min in one eye and LASIK in the fellow eye. The 1-year follow-up was evaluated.

**Results:**

There were no differences between LASIK and MMC-PRK eyes preoperatively. Forty-two patients completed the 1-year follow-up. MMC-PRK eyes achieved better uncorrected visual acuity (p = 0.03) and better best-spectacle-corrected visual acuity (p<0.001) 1 year after surgery. SE did not differ in the two groups during follow-up (p = 0.12). Clinically significant haze was not found in surface ablation eyes. LASIK eyes showed a greater higher-order aberration (p = 0.01) and lower contrast sensitivity (p<0.05) than MMC-PRK eyes postoperatively. Excellent vision was reported in 64% of LASIK and 74% of MMC-PRK eyes 1 year after surgery. The corneal resistance factor and corneal hysteresis (ORA, Reichert) were higher in LASIK than in MMC-PRK eyes (p<0.01) at the last follow-up.

**Conclusions:**

Wavefront-guided PRK with 0.002% MMC was more effective than wavefront-guided LASIK for correction of moderate myopia. Further research is necessary to determine the optimal concentration, exposure time and long-term corneal side effect of MMC.

Excimer laser photorefractive keratectomy (PRK) with adjunctive mitomycin C (MMC; MMC–PRK) has recently been used as an alternative to laser in situ keratomileusis (LASIK) for surgical correction of refractive errors.^1^ ^2^ ^3^ Although surface ablation usually has a slower visual recovery and more early postoperative discomfort, it avoids LASIK flaps complications and possibly results in less corneal biomechanical instability.^4^ ^5^

Mitomycin C is an alkylating agent that inhibits DNA and RNA replication and protein synthesis.^6^ It regulates fibroblast proliferation and differentiation, and subsequently blocks myofibroblast formation, which is responsible for corneal haze after PRK in high myopic corrections.^7^ ^8^ Recent studies have shown that low-dose MMC (0.002%) has a similar efficacy to standard MMC concentration (0.02%) in preventing postoperative haze following surface ablation for moderate myopia corrections, and also minimise potential side effects.^7^ ^9^ ^10^

There are not many papers in the literature comparing MMC-PRK and LASIK. Randleman *et al*^3^ compared wavefront-optimised PRK with standard dose MMC and wavefront-optimised LASIK in 272 preoperative refraction-matched eyes for moderate myopia corrections. They found a better uncorrected visual acuity (UCVA) and spherical equivalent (SE) in MMC-PRK eyes 3 months after surgeries.

The purpose of this study is to compare visual acuity (VA) outcomes (including satisfaction questionnaire, aberrometry, contrast sensitivity) and corneal biomechanical properties 1 year after wavefront-guided PRK with 0.002% MMC and LASIK for myopic corrections. As a continuum of our early postoperative outcomes study,^11^ we are unaware of any randomised prospective study in the literature comparing 1-year results of PRK with MMC and LASIK consecutively performed in both eyes of the same patients at the same treatment sitting.

## Patients and methods

### Study design and patient selection

Forty-four patients (88 eyes) with myopic astigmatism and an estimated ablation depth greater than 50 μm using the LADARWave 4000 (Alcon Laboratories, Fort Worth, Texas) platform in both eyes (OU) were randomised to receive wavefront-guided PRK with prophylactic application of MMC 0.002% (0.02 mg/ml) in one eye and wavefront-guided LASIK in the fellow eye. The right eye of each patient was randomised at the surgical centre using a coin toss to either one of the procedures; the other eye automatically received the other technique. The ablation was calculated with an optical zone (OZ) of 6.5 mm diameter and transition zone (TZ) of 1.25 mm.

The inclusion criteria were best-spectacle-corrected visual acuity (BSCVA) of logMAR 0.0 (Snellen 20/20) or better in OU, at least 6 months’ refraction stability, an estimated residual corneal ultrasound pachymetry greater than 410 μm in OU and a complete ophthalmological exam without associated diseases.

The exclusion criteria included EyeSys 2000 (EyeSys, Houston, Texas) and/or Orbscan II (Orbtek/Bausch & Lomb, Munich, Germany) topographic patterns suggestive of ectatic disease or disease status that could interfere with the healing process of the cornea, that is, collagen vascular disease, diabetes. Patients with a history of severe ocular trauma or previous ocular surgery were also excluded.

Approval for the study was granted by the Institutional Review Board of the Federal University of São Paulo, Brazil, and the study was conducted in accordance with the tenets of the Declaration of Helsinki. All patients provided informed consent, and the Clinical Trial Registration was carried out at http://www.clinicaltrials.gov at code NCT 00365040.

### Surgical procedures

After topical anaesthesia, the central 9 mm diameter epithelium was removed mechanically using a scarificator blade in all PRK eyes. Custom ablation (OZ 6.5 mm, TZ 1.25 mm) was then performed with the LADARWave 4000 laser. Following photoablation, 0.002% MMC was applied to the stromal bed for 1 min. The solution was applied by filling the barrel of a 7.0 mm Hoffer marking trephine centred over the pupil. The MMC was dried after 60 s using a sterile microsponge. The eye was then copiously irrigated with 30 cm^3^ of balanced salt solution to wash out residual MMC. A bandage contact lens (New Vues, CIBA, Duluth) was placed at the end of the procedure.

After topical anaesthesia and corneal marking, the LASIK flap was cut using a Moria M2 (Moria, Antony, France) microkeratome. “Ring and stop” was chosen accordingly to achieve a 9 mm diameter flap. Custom ablation was then performed. Corneal irrigation and flap repositioning were done following photoablation.

No offset or laser nomogram adjustment was used in the two groups. Surgeries were performed by both authors.

All operated eyes received the same postoperative drug regimen. Tobramycin 0.3% plus dexamethasone 0.1% drops were given four times daily for 15 days; artificial tears were given five times daily for at least 3 months. Patients were instructed to take pain-relief tablets if necessary. The bandage contact lens in PRK eyes was removed after complete corneal reepithelialisation.

No enhancements were performed in any eye during 12 months’ follow-up.

### Patient assessment

The preoperative visit involved a comprehensive ophthalmological examination including UCVA, BSCVA, cycloplegic refraction, corneal topography, aberrometry, central ultrasound pachymetry (USP), slit-lamp microscopy and contrast sensitivity. Follow-up examinations were scheduled at 1, 3, 6 and 12 months after surgery, and involved the same tests and assessments performed during the preoperative visit.

UCVA and BSCVA were measured using the Early Treatment Diabetic Retinopathy Study visual acuity chart (ETDRS, logMAR notation). Cycloplegic refraction and aberrometry (LADARWave 4000, Alcon Laboratories) were performed 40 min after two drops (5 min apart) of 1% cyclopentolate. All aberrations were measured to the fifth Zernike order using a 6.5 mm pupil.

At each follow-up, a satisfaction questionnaire was administered prior to any other testing. Patients were asked to assess their vision in each eye as bad, reasonable, good or excellent; they were also asked to rate ocular pain, far vision difficulty, near vision difficulty, glare, photophobia, vision fluctuation, image distortion and foreign-body sensation in each eye. Each characteristic was grade on a 0 to 3 scale, with 0 indicating an absence of symptoms and 3 indicating the worst symptom.

Contrast sensitivity (Optec 6500, FACT, Stereo Optical Co, Chicago) was determined in each eye with the BSCVA at spatial frequencies of 1.5, 3, 6, 12 and 18 cycles per degree in mesopic and photopic conditions. The log base 10 contrast sensitivity values were used to construct a graph for each spatial frequency tested.

Biomechanical properties of the cornea were determined using the Ocular Response Analyser (ORA, Reichert, Depew, New York) at 1-year follow-up. Corneal hysteresis (CH) and corneal resistance factor (CRF) were measured in both eyes of patients.

Statistical analysis was done using analysis of variance with repeated measures (over time and between treatments, Bonferroni) using the statistics software SPSS 12 (SPSS, Chicago). The definition of statistical significance was set at p<0.05.

## Results

Forty-four patients (88 eyes) were enrolled in this study. The mean age in both groups was 31.7 years, range 21 to 54 years old. There were 26 females (59%) and 18 (41%) males. The mean UCVA, mean BSCVA and mean SE correction before surgery were 1.23 (SD 0.15) (logMAR notation), −0.09 (0.07) (logMAR) and −3.99 (1.20) D, respectively in LASIK eyes and 1.21 (0.15) (logMAR), −0.08 (0.08) (logMAR) and −3.85 (1.12) D, respectively in surface ablation eyes (p>0.05). The mean preoperative USP was 542.8 (25) μm in LASIK eyes and 544.7 (25.5) μm in MMC-PRK eyes (p = 0.013). The mean higher-order aberration (HOA) was 0.39 (0.16) μm in LASIK and 0.38 (0.13) μm in MMC-PRK eyes (p>0.05). The mean ablation depth (AD) was 73.09 (14.55) μm in LASIK eyes and 70.7 (14.07) μm in MMC-PRK eyes (p = 0.074). There was also no statistically significant between-group difference in contrast sensitivity before surgery (p>0.05). Forty-two patients completed 1-year follow-up.

**Figure 1:**
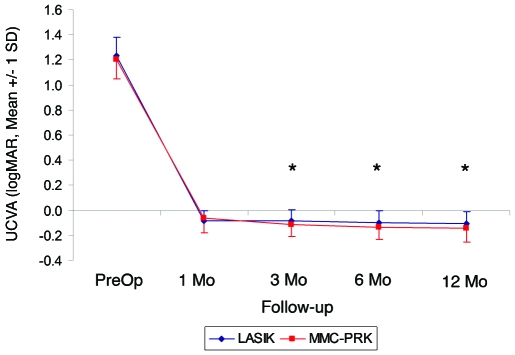
Uncorrected visual acuity (UCVA, logMAR notation, mean and SD) in laser in situ keratomileusis (LASIK) and photorefractive keratectomy with mitomycin C (MMC-PRK) eyes during 1-year follow-up. A UCVA of −0.2 or better (Snellen 20/12.5) was achieved in 52% of eyes after MMC-PRK compared with 31% of eyes after LASIK at the last visit (p = 0.027). *Statistically significant results.

**Figure 2:**
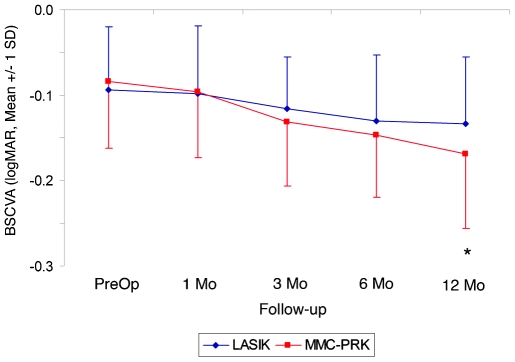
Best spectacle-corrected visual acuity (BSCVA, logMAR notation, mean and SD) in laser in situ keratomileusis (LASIK) and in photorefractive keratectomy with mitomycin C (MMC-PRK) eyes during the 1-year follow-up. At 1 year postoperatively, significantly more eyes in the surface ablation group gained one or more lines (74% in MMC-PRK eyes vs 43% in LASIK eyes, p<0.001).

The UCVA in the two groups during follow-up is summarised in fig 1. The MMC-PRK group achieved statistically significant better mean values 3, 6 and 12 months after surgery. The mean BSCVA results in the two groups are shown in fig 2. MMC-PRK eyes had statistically significant better results at the 1-year follow-up. There was no statistically significant difference in mean SE in the two groups during 1-year follow-up (p = 0.116). At the last follow-up, the mean SE was 0.45 (0.54) D in LASIK and 0.48 (0.38) D in MMC-PRK eyes.

No surface ablation eye presented a clinically significant haze (more than grade 1 haze with the Fantes^12^ scale during follow-up).

The total root-mean-square (RMS), defocus, astigmatism, HOA and spherical aberration were statistically significantly higher in LASIK eyes than in the MMC-PRK group during the 1-year follow-up (p<0.05). Other higher-order aberrations, up to fifth-order aberrations excluding coma and spherical aberration, were also higher in the LASIK group 3, 6 and 12 months after surgery (p<0.01). HOA, coma (Z 3) and spherical aberration (Z 4,0) showed a statistically significant increase in both groups postoperatively. Table 1 lists the magnitude of wavefront aberrations until the fifth order for a 6.5-mm pupil in both groups at 12 months’ follow-up.

**Table 1:** Wavefront analysis using a 6.5 mm pupil diameter (mean and SD) in laser in situ keratomileusis (LASIK) and in fellow photorefractive keratectomy with mitomycin C (MMC-PRK) eyes 1 year after surgery

	Total RMS*	Defocus*	Astigmatism*	HOA*	Coma	Spherical*	Other*
LASIK	1.15 (0.37)	0.75 (0.45)	0.36 (0.29)	0.66 (0.19)	0.32 (0.17)	0.43 (0.20)	0.33 (0.13)
MMC-PRK	0.91 (0.30)	0.57 (0.35)	0.25 (0.18)	0.58 (0.24)	0.29 (0.20)	0.39 (0.21)	0.26 (0.12)

HOA, higher-order aberrations until the fifth order; Other, higher-order aberrations until the fifth-order RMS other than coma and spherical aberration; RMS, root mean square; Spherical, spherical aberration.

*Statistically significant differences between groups. Forty-two patients completed follow-up.

**Figure 3:**
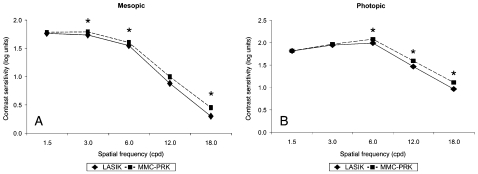
Contrast sensitivity (log units) at spatial frequencies of 1.5, 3.0, 6.0, 12.0 and 18.0 in laser in situ keratomileusis (LASIK) and photorefractive keratectomy with mitomycin C (MMC-PRK) eyes at mesopic (A) and photopic (B) conditions 1 year after surgery. *Statistically significant results.

Figure 3 shows the contrast sensitivity in photopic and mesopic conditions in the two groups 1 year after surgery. MMC-PRK eyes presented statistically significant better mesopic contrast sensitivity at spatial frequencies 3, 6 and 18 cpd during the 1-year follow-up. Surface ablation eyes also showed a statistically significant better photopic contrast sensitivity than LASIK eyes at spatial frequencies of 6, 12 and 18 cpd postoperatively.

One year after surgery, excellent vision was reported in 64% and 74% of LASIK and MMC-PRK eyes respectively. Far-vision difficulty, glare and vision fluctuation were also more frequently reported in LASIK than in MMC-PRK eyes at last follow-up. Foreign-body sensation was more prevalent in MMC-PRK eyes 1 year after surgery.

Corneal resistance factor (CRF) and corneal hysteresis (CH) were statistically significantly higher in the LASIK group than in MMC-PRK eyes 1 year after surgery (p<0.01, table 2). The central ultrasound pachymetry was statistically significant lower in PRK with MMC eyes in all postoperative examinations.

**Table 2:** Corneal biomechanical properties using the Ocular Response Analyser in laser in situ keratomileusis (LASIK) and photorefractive keratectomy with mitomycin C (MMC-PRK) eyes 1 year after surgery

	LASIK	MMC-PRK	p Value
CRF	7.33 (0.98)	7.05 (1.11)	0.018
CH	8.55 (0.95)	8.11 (1.09)	0.001

CH, corneal hysteresis; CRF, corneal resistance factor.

## Discussion

Recently, there has been renewed interest in advanced surface ablation which includes PRK with MMC, laser subepithelial keratectomy and epi-LASIK. Although associated with a longer visual rehabilitation period, surface ablation techniques are less related to initial or secondary flap complications, including ectasia.^5^

PRK for correcting intermediate to high levels of myopia may result in a strong wound-healing reaction, leading to haze formation and suboptimal refractive outcomes. We chose to use intraoperative topical application of MMC in a millesimal concentration (0.002%) during PRK based on Netto *et al*’s study.^7^ These lower concentrations of MMC have a similar efficacy to higher concentrations in reducing haze, but also minimise potential side effects. Thornton *et al*^9^ ^10^ reported two studies with surface ablation and low-dose mitomycin C. In the first study,^9^ they found that low-dose MMC (0.002%) after laser epithelial keratomileusis (LASEK) for correction of moderate and high myopia results in less corneal haze than in eyes not receiving this agent. In the second study,^10^ the authors retrospectively compared the lower dose MMC with that of the standard dose (0.02%) in eyes treated with PRK for myopia. They found that MMC 0.02% is more effective than low-dose MMC in preventing postoperative haze following surface ablation for myopia greater than −6.00 D and an ablation depth greater than 75 μm. However, for moderate myopia and shallow depth, the authors found both MMC concentrations to be equally effective. The concern for mitomycin C use stems from complications arising in scleral and corneoscleral procedures with mitomycin C, including peripheral keratolysis and scleral melting.^13^ Although these effects have not been shown to occur in cases of topical MMC use during surface refractive surgery, some concern still exists for MMC long-term toxicity to keratocytes, endothelial cells, and intraocular structures.^7^ ^8^ Some studies have reported a decrease in endothelial cell count and detection of MMC in the anterior chamber in eyes that had received MMC after surface laser ablation.^14^ ^15^ ^16^

This study suggests a potential advantage for surface ablation over LASIK at the 1-year follow-up. At 12 months postoperatively, UCVA and BSCVA were better in MMC-PRK eyes. More eyes achieved the supranormal UCVA of −0.2 or better in MMC-PRK (52%) than in LASIK (31%) eyes at the 1-year follow-up. Seventy-four per cent of MMC-PRK and 43% of LASIK eyes gained one or more BSCVA lines at 12 months postoperatively. There were no differences in SE at last follow-up, and both groups presented a mean hyperopic shift around 0.5 D 1 year after surgery. However, MMC-PRK treatments appeared more precise (lower SDs in MMC-PRK eyes), and more eyes were myopic after LASIK surgery and hyperopic after MMC-PRK. Nomogram adjustments should improve refractive outcomes in both groups and might reduce the UCVA advantage of MMC-PRK. Our findings are similar to those of Randleman *et al.*^3^ In a retrospective study comparing 272 refraction-matched eyes that had undergone MMC-PRK or LASIK, they found significantly better results in surface-ablation eyes 3 months after wavefront-optimised surgeries.

In our study, we found better aberration outcomes in MMC-PRK eyes. Total RMS, HOA, defocus, astigmatism, spherical aberration and other aberrations were lower in eyes that had received MMC-PRK surgery. We conjecture that the absence of a flap interface and the modulation of corneal wound healing with MMC resulted in less induction of aberrations. It is unclear whether the decreases in aberrations are clinically relevant, but MMC-PRK eyes had better refractive outcomes, had better contrast sensitivity scores and were better rated in terms of visual satisfaction. Porter *et al*^17^ reported a significant increase in higher-order aberrations of approximately 30% 2 months after cutting a Hansatome (Bausch & Lomb, Rochester, New York) microkeratome LASIK flap without laser application. Pallikaris *et al*^18^ also found a significant increase in total higher-order wavefront aberrations following flap formation. Some studies have demonstrated that femtosecond laser flap formation results in a smaller increase in higher-order aberrations than mechanical flap creation.^19^

Several authors have reported significant correlations between increased higher-order aberrations and decreased contrast sensitivity, especially total HOA, coma and spherical aberrations.^20^ ^21^ Other authors have reported the correlation between visual symptoms and ocular aberrations, such as monocular diplopia with coma, and starburst and glare with spherical aberration.^21^ ^22^ In the present study, MMC-PRK eyes had lower aberrations and better mesopic and photopic contrast sensitivity scores than LASIK eyes. Surface ablation eyes scored higher in terms of the visual-satisfaction questionnaire.

Corneal hysteresis and corneal resistance factor are biomechanical properties of the cornea which reflect its viscoelastic properties. Corneal hysteresis has been previously shown to decrease following LASIK surgery.^23^ Corneal hysteresis and corneal resistance factor values also have been shown to be significantly decreased in keratoconic eyes.^23^ ^24^ We found a statistically significant difference in corneal hysteresis and corneal resistance factor between LASIK and MMC-PRK eyes 1 year after surgeries. In our study, eyes that had received MMC during PRK showed a lower CH and CRF than LASIK eyes. One weakness of our study is that we did not measure preoperative corneal hysteresis and corneal resistance factor in both groups. Kirwan and O’Keefe^25^ found a statistically significant decrease in hysteresis 3 months after LASIK and LASEK with similar decrements in both treatments groups. The authors also found a moderately strong correlation between central corneal thickness (CCT) and hysteresis. In our study, MMC-PRK eyes had statistically significant thinner corneal measures than LASIK eyes 1 year after surgeries. We are not sure whether the lower hysteresis values found in MMC-PRK eyes are related to reduced biomechanical integrity of the cornea or are a result of the lower postoperative CCT found in PRK with MMC eyes. Future studies are needed to determine the Ocular Response Analyser accuracy to measure the biomechanical properties of the cornea and its clinical relevance.

In the current study, wavefront-guided PRK with 0.002% MMC was more effective than wavefront-guided LASIK for correction of moderate myopia during the 1-year follow-up. Surface ablation eyes presented a better UCVA, BSCVA, aberrometry and contrast sensitivity, and were better rated in a subjective questionnaire than LASIK eyes. However, before widespread use of prophylactic 0.002% MMC can be implemented, further research is necessary to determine the optimal concentration and exposure time, and the long-term corneal side effect of mitomycin C exposure.
